# 2253: An analysis of how consumer physical activity monitors (monitors) are used in biomedical research

**DOI:** 10.1017/cts.2017.148

**Published:** 2018-05-10

**Authors:** Stephen P. Wright, Kathryn Sandberg

## Abstract

OBJECTIVES/SPECIFIC AIMS: To analyze how consumer physical activity monitors are currently used in biomedical research. METHODS/STUDY POPULATION: Searches were conducted in Ovid Medline, PubMed Medline, clinicaltrials.gov, and NIH RePORTER using search terms including Fitbit, Jawbone, Apple watch, Garmin, Polar, Microsoft band, Misfit, Nike, Withings, and Xiaomi. Results were quantitated by category: condition/topic, intervention, enrollment status, study type and design, age, grant mechanism, and primary outcome. RESULTS/ANTICIPATED RESULTS: Fitbit is used >80%. There are 127 clinical studies using Fitbit devices listed in clinicaltrials.gov. In total, 48 have been completed while 79 are ongoing. Some studies have already published their findings; 40 papers cited in Ovid MEDLINE report use of a Fitbit device. NIH is now funding research that uses consumer physical activity monitors, and the NIH RePORTER shows the number of grants using Fitbit is rapidly increasing. DISCUSSION/SIGNIFICANCE OF IMPACT: The current state and potential growth of this technology is transforming biomedical research and is enabling us to ask new and more granular questions about activity and sleep in health and disease.

